# Studying large biomolecules as sedimented solutes with solid-state NMR

**DOI:** 10.52601/bpr.2024.240014

**Published:** 2024-08-31

**Authors:** Fan Shi, Tong Zhang, Juan Li, Chaowei Shi, Shengqi Xiang

**Affiliations:** 1 MOE Key Lab for Cellular Dynamics, School of Life Sciences, Division of Life Sciences and Medicine, University of Science and Technology of China, Hefei 230026, China; 2 Hefei National Laboratory for Physical Sciences at Microscale, University of Science and Technology of China, Hefei 230026, China

**Keywords:** Sedimentation NMR, Solid-state NMR, Ultracentrifugation, Magic angle spinning, Nucleosome

## Abstract

Sedimentation solid-state NMR is a novel method for sample preparation in solid-state NMR (ssNMR) studies. It involves the sedimentation of soluble macromolecules such as large protein complexes. By utilizing ultra-high centrifugal forces, the molecules in solution are driven into a high-concentrated hydrogel, resulting in a sample suitable for solid-state NMR. This technique has the advantage of avoiding the need for chemical treatment, thus minimizing the loss of sample biological activity. Sediment ssNMR has been successfully applied to a variety of non-crystalline protein solids, significantly expanding the scope of solid-state NMR research. In theory, using this method, any biological macromolecule in solution can be transferred into a sedimented solute appropriate for solid-state NMR analysis. However, specialized equipment and careful handling are essential for effectively collecting and loading the sedimented solids to solid-state NMR rotors. To improve efficiency, we have designed a series of loading tools to achieve the loading process from the solution to the rotor in one step. In this paper, we illustrate the sample preparation process of sediment NMR using the H1.4-NCP^167^ complex, which consists of linker histone H1.4 and nucleosome core particle, as an example.

## INTRODUCTION

Nuclear magnetic resonance (NMR) technology has a wide range of applications in the fields of chemistry, biology, and medicine. In structural biology, NMR is one of the few techniques that can provide information with atomic resolution. However, the commonly used liquid-state NMR methods require samples to be in a homogeneous solution. As the molecular weight of the target molecule increases, the signals broaden due to slower molecular motion, which limits the applicability of this technique. In contrast, the linewidth of solid-state NMR (ssNMR) signals is theoretically independent of the size of the molecule, overcoming the molecular weight limitations of liquid-state NMR in protein research (Demers *et al.* 2018). The application of Magic-Angle Spinning (MAS), Cross-Polarization(CP), and high-power decoupling has made ssNMR increasingly popular (Reif *et al.* 2021). The development of ultrafast magic-angle spinning technology has significantly improved the sensitivity and resolution of solid-state NMR (Nishiyama *et al.* 2022). Consequently, ssNMR is becoming more favored in the research of related biological problems. The typical research objects of ssNMR are mainly solid-state biomolecule microcrystals (Castellani *et al.* 2002), protein fibers (Tycko 2011), and membrane proteins embedded in phospholipids (Gopinath *et al.* 2021). Biomacromolecules in the solution state cannot be measured directly and must be converted into solid by some methods, usually crystallization (Ahlawat *et al.* 2022).

In recent years, sedimentation NMR methods have emerged as a way to extend solid-state NMR methods to soluble biomolecules. The sedimentation NMR methods separate soluble macromolecules from the initial solution by centrifugation to form a gel-like sediment for use in ssNMR experiments. Unlike precipitates, sedimental samples rely on sufficiently strong centrifugal force applied for an extended period. Sedimental samples need not be crystallized or fibrillated to convert the soluble proteins into a "solid" state. The entire process does not involve chemical treatment and maintains the biological activity of the sample. The concept of sedimentation NMR originated from the pioneering work of Bernd Reif, who first observed the phenomenon of sample sedimentation due to strong centrifugal forces in solid-state NMR experiments (Mainz *et al.* 2009). Subsequently, Bertini Ivano and his team further refined this concept, demonstrating through a series of experiments the advantages of sedimentation NMR in maintaining the native state of samples and enhancing NMR signal resolution (Bertini *et al.* 2011, 2013b). Notably, the sediment process is a physical transformation that does not depend on the specific biochemical properties of each biomacromolecule. Therefore, it is a universal method applicable to biomacromolecules with molecular weights greater than 100 kDa, and examples with smaller molecular weights also exist (Bell *et al.* 2024; Fragai *et al.* 2013; Stöppler *et al.* 2018). The spectra line widths of sedimental samples are comparable to those of samples in crystalline or fibrous samples, likely due to the alignment of the molecules in their orientation during the sedimentation process (Bertini *et al.* 2013b). Thanks to the advantages of ssNMR being not limited by molecular weight and the introduction of the sedimental method, high molecular weight biomolecules such as enzymes, molecular machines, and large protein complexes can be directly studied using solid-state NMR (Gardiennet *et al.* 2012; Gauto *et al.* 2019; Klein *et al.* 2022; Stanek *et al.* 2020). Here, in Table 1 and Table 2, we have listed some representative results on solid-state NMR application using *in situ* and *ex situ* sedimentation NMR, with references for further reading.

**Table 1:** Some representative results of solid-state NMR applications using *In situ* sedimentation NMR

Molecules	MW	Rotor size	Spin rate	Reference
Ferritin	20 kDa × 24	N/A	N/A	Rothen 1944
		4 mm	3 kHz	Bertini *et al.* 2011
		1.3 mm	60 kHz	Bertini *et al.* 2012b
αB-crystallin	20 kDa × 30	4 mm	12 kHz	Mainz *et al.* 2009
		3.2 mm	8 kHz	Sarkar *et al.* 2016
		3.2 mm	12 kHz	Baldwin *et al.* 2012
Bovine serum albumin (BSA)	68 kDa	3.2 mm	14 kHz	Bertini *et al.* 2012b
Carbonic anhydrase	29 kDa	4 mm	12 kHz	Bertini *et al.* 2012b
Met-0 Aβ40	4.6 kDa × 15	4 mm	12 kHz	Bertini *et al.* 2013a
hSOD	32 kDa × 2	3.2 mm	14 kHz	Fragai *et al.* 2013
Proteasome (11S-α7β7β7α7-11S)	1.1 MDa	3.2 mm	22 kHz	Mainz *et al.* 2013

**Table 2:** Some representative results of solid-state NMR applications using *ex situ* sedimentation NMR

Molecules	MW	Centrifuge force	Centrifuge time	Reference
dodecameric helicase (DnaB)	59 kDa × 12	200,000 *g*	overnight	Gardiennet *et al.* 2012
		210,000 *g*	16 h	Wiegand *et al.* 2016
		200,000 *g*	16 h	Wiegand *et al.* 2018
Met-0 Aβ40	4.6 kDa × 15	N/A	N/A	Bertini *et al.* 2013a
Ubiquitin	8.6 kDa × 2	32,000 r/min	6 days	Fragai *et al.* 2013
AP205 (icosahedral particle)	2.5 MDa	165,000 *g*	15 h	Barbet-Massin *et al.* 2014
The neonatal Fc receptor (FcRn)	42 kDa	100,000 *g*	40 h	Bell *et al.* 2024; Stöppler *et al.* 2018
Rpo7/4	33.5 kDa	35,000 r/min	16 h	Torosyan *et al.* 2019
pRN1 + DNA	40 kDa + 5.4 kDa	210,000 *g*	16 h	Boudet *et al.* 2019
Nucleosomes + LANA	210 kDa + 2 kDa	N/A	18 h	Xiang *et al.* 2018
Nucleosomes + PHD2	208 kDa + 6 kDa	83,000 *g*	24–28 h	le Paige *et al.* 2021

**Figure 1:**
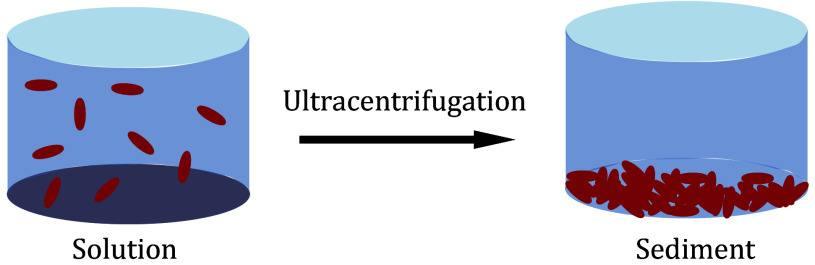
Sedimentation driven by centrifugal force

In order to determine the appropriate centrifuge force, as shown in Fig. 1, researchers can utilize the provided formula in the references to calculate the distribution of protein under a specific centrifuge force (Bertini *et al*. 2011). Alternatively, this document (Cole *et al*. 2008) offers insights into the centrifugal forces necessary for sedimentation velocity experiments across a wider molecular weight range, providing a valuable reference for estimating the centrifugal forces used in sediment NMR. Moreover, if analytical ultracentrifugation (AUC) equipment is accessible, researchers can conduct preliminary assessments using sedimentation velocity or sedimentation equilibrium experiments to ascertain the appropriate centrifuge force and sedimentation time.

The magic angle spinning (MAS) is commonly employed in modern solid-state nuclear magnetic resonance (ssNMR) studies, in which the samples are loaded into cylindrical rotors with outer diameters ranging from 7 to 0.7-mm. However, loading biological samples into the rotor has always been challenging. Any losses at this stage are particularly damaging since the sample lost during the loading step is the final pure sample, which must be minimized (Lacabanne *et al.* 2019). Typically, ssNMR sample loading is performed in two separate steps: first, the sample is spun down, and then the pellets are transferred to the rotor. There are two primary methods of packing the sample. One such method is using the sample loading toolset provided by Bruker, which is more suitable for loading dry powder samples. However, this method often results in a significant amount of sample adhering to the loading tool when the water content and viscosity of biological samples are high, leading to losses.

Additionally, when filling samples with high water content, it becomes difficult to use a pestle and mortar to compact the sample, resulting in loosely packed samples. Consequently, the actual amount of loaded sample is small, leading to weak signals. Repeated filling is often required if the sample viscosity is too high, leading to prolonged loading times and a higher risk of sample dehydration and inactivation.

Also, some laboratories transfer the sediment to the rotor using a funnel-shaped device made from a pipette tip combined with a fixed-angle benchtop centrifuge (Bertini *et al.* 2012a). While this method can be applied to samples with some viscosity, it is cumbersome and heavily relies on the operator's experience. Moreover, this method is not standardized, and there is still a risk of sample loss or partial dehydration during transfer because the viscous sample "choked" the pipette. As the size of the rotor decreases, the method becomes less convenient to operate and carries a higher corresponding risk. This method is challenging to implement in small-diameter MAS rotors (*e*.*g*., 1.3-mm and 0.7-mm) because such rotors have small diameters and thin wall thicknesses, which can easily damage the rotor during sample transfer. In ssNMR studies, using small-sized rotors is becoming increasingly popular with the maturity of hydrogen detection methods (Le Marchand *et al.* 2022). In summary, the existing ssNMR loading methods have a number of drawbacks, including significant sample losses during transfer, inconvenient operation, risk of sample dehydration and inactivation, and unsuitability for small rotors.

In solid-state NMR (ssNMR), sediment NMR samples require longer centrifugation times and possess higher viscosity and water content than regular ssNMR samples. Therefore, a one-step deposit of biological samples directly from the solution into the NMR rotor is an optimal approach that avoids sample loss and dehydration caused by additional operations and transfers. This process requires specialized tools, and previous studies have developed specific loading tools to deposit biomolecules into ssNMR rotors (Böckmann *et al.* 2009; Mandal *et al.* 2017). The one-step centrifugation results in a compact sample that is uniformly distributed in the rotor, contributing to the rotor's stability during spinning. However, the development of solid-state NMR sample loading tools needs further optimization and improvement to enhance the success rate of solid-state NMR experiments, particularly for small rotors, which are scarce in the market. Thus, designing an efficient and reproducible loading scheme for biological samples by developing corresponding tools is imperative. Our laboratory has developed a set of sample packing tools for ssNMR that can withstand prolonged ultracentrifugation and are suitable for a wide range of Bruker solid rotors.

The sedimentation NMR method has opened up new avenues for studying high molecular weight targets, such as nucleosomes, by solid-state NMR, thanks to the development of appropriate loading tools. Nucleosomes, which are fundamental components of chromatin and important protein–DNA complexes, consist of eight histones and a segment of 147 bp DNA, resulting in a molecular weight of approximately 210 kDa, fully compatible with sediment NMR. Using nucleosome samples obtained through sediment NMR, high-resolution NMR spectra of the histone proteins can be obtained, enabling the study of nucleosomes (Xiang *et al.* 2018). This approach provides a powerful tool for investigating the fundamental biological processes that involve nucleosomes and has the potential to shed light on the molecular mechanisms underlying various cellular processes (van Emmerik and van Ingen 2019).

## OVERVIEW OF PROTOCOL

**Figure 2:**
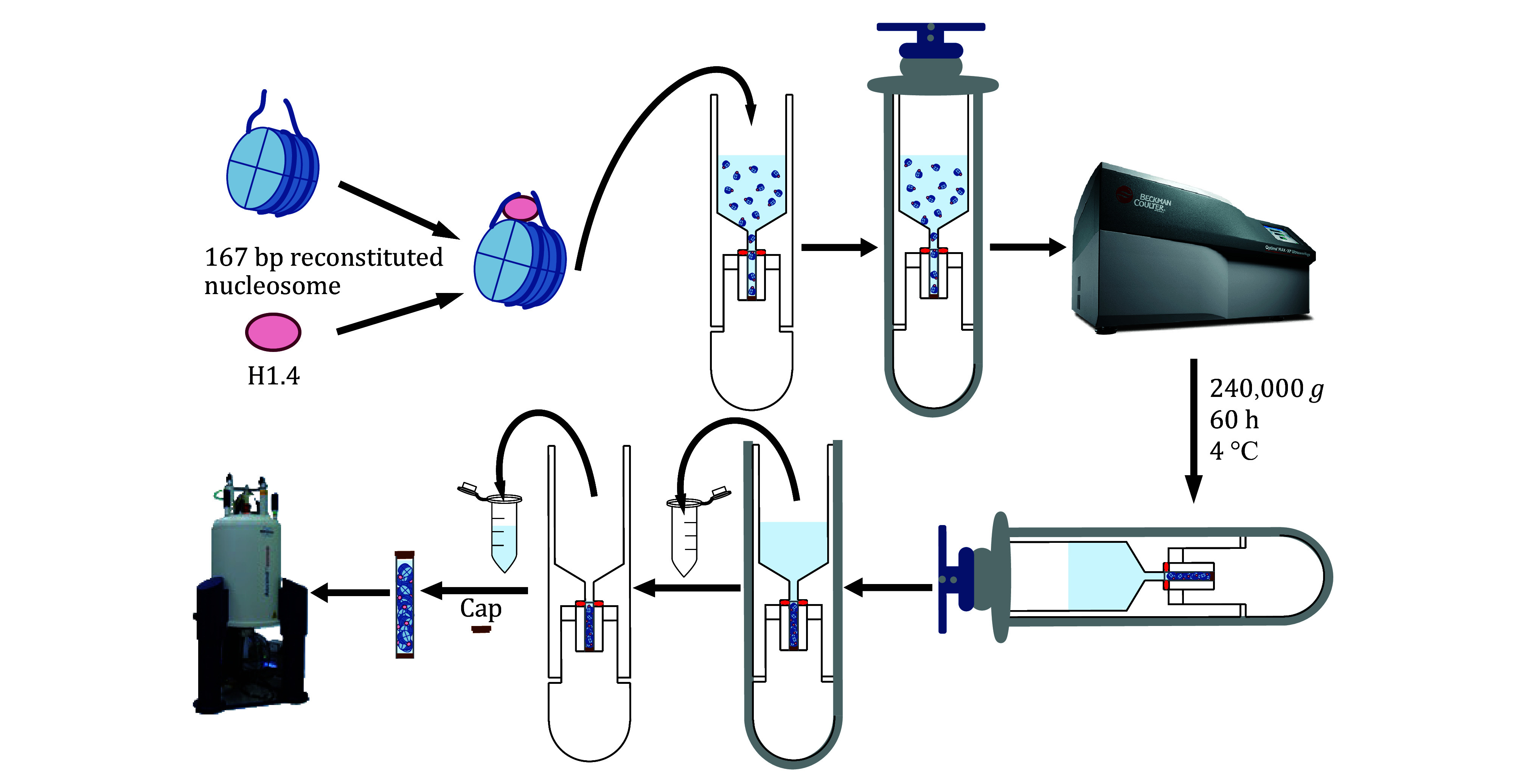
Technology roadmap of H1.4-NCP^167^ by sediment NMR

The preparation of high-quality solid-state NMR samples is a crucial step in obtaining reliable and informative NMR spectra. This study presents a simple and efficient method for preparing solid-state NMR samples of the H1.4-NCP^167^ nucleosome complex (Fig. 2), starting from samples initially in solution. First, the H1.4-NCP^167^ nucleosome complex is prepared in solution. Next, a suitable solid-state NMR rotor loading tool is employed to transfer the solution-state H1.4-NCP^167^ complex to a 1.3-mm solid-state NMR MAS rotor. Finally, the loaded rotor is capped, and the sample is subjected to solid-state NMR measurements on a solid-state NMR spectrometer. This approach offers several advantages over traditional solid-state NMR sample preparation methods, including a single-step conversion of samples from solution to solid-state, minimal sample loss, and preserving sample integrity during transfer. In summary, this method provides a simple and efficient means of preparing solid-state NMR samples for subsequent studies of the H1.4-NCP^167^ nucleosome complex, which also has the potential to extend to other soluble complexes.

## EXPERIMENTAL DESIGN

### Sample preparation

The sample preparation scheme is based on the previously established methods (le Paige *et al.* 2021; Luger *et al.* 1999; Song *et al.* 2014), in which the technical details can be found.

#### Expression and purification of human histone H1.4

The full-length H1.4 protein is first purified using nickel affinity chromatography and followed by cation exchange chromatography.

#### Expression and purification of Xenopus laevis histones

The individual histones are expressed in inclusion bodies. The purification of histones was carried out under denaturing conditions. We use gravity columns for cation exchange chromatography and C4 columns for reverse-phase chromatography to obtain the four histones (H2A, H2B, H3, H4), followed by lyophilization.

#### Preparation of 167 bp DNA

The plasmid PWM530 containing 12 × 167 bp Widom 601 was transformed into competent DH5α cells. Plasmid DNA was extracted on a large scale, followed by enzymatic digestion, PEG precipitation, and anion exchange chromatography to obtain milligram quantities of the 167 bp DNA fragment.

#### Preparation of the nucleosome core particle (NCP) complexed with histone H1.4 (H1.4-NCP^167^)

**Figure 3:**
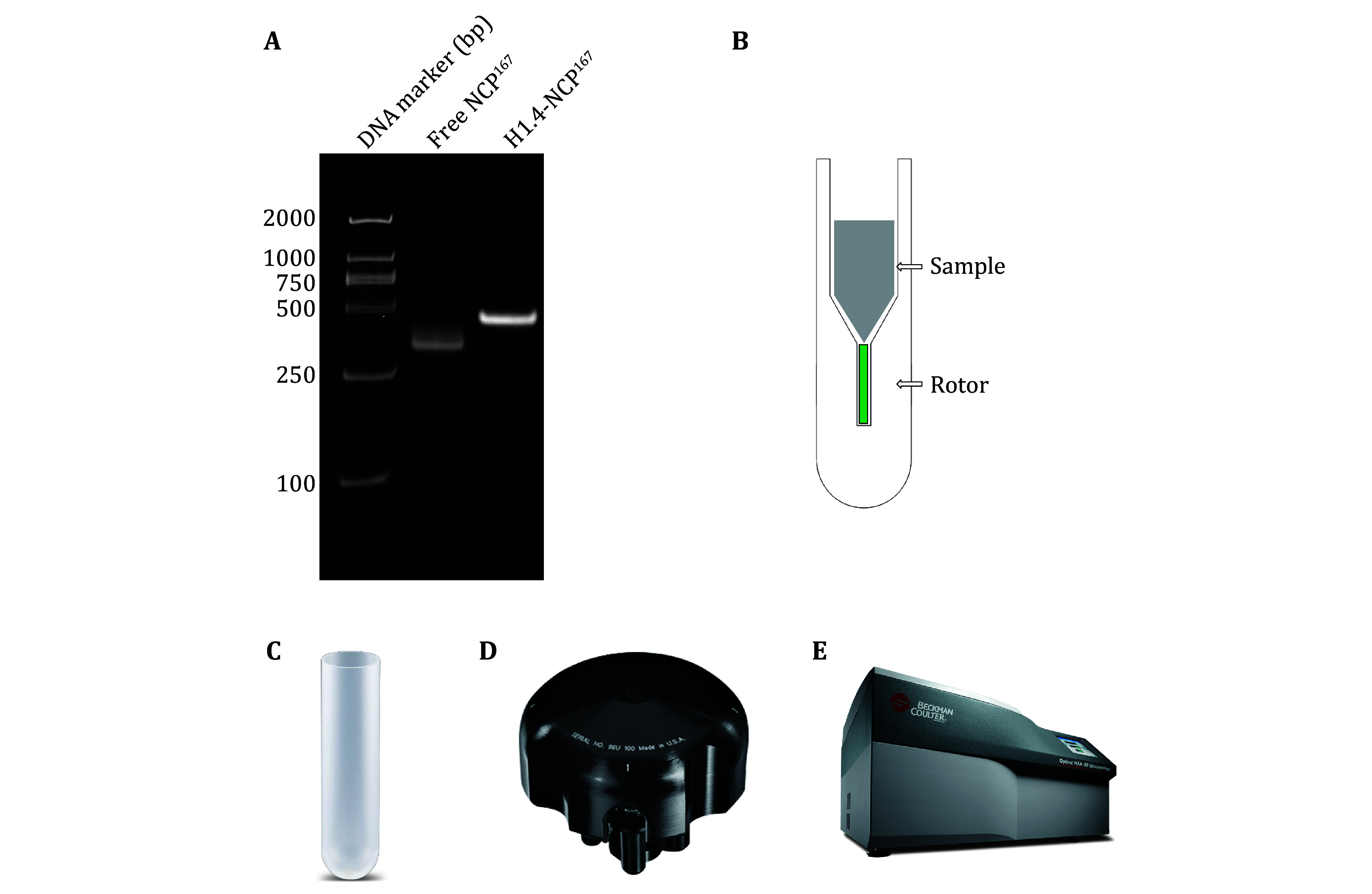
**A** Preparation of nucleosome complex H1.4-NCP^167^. Electrophoresis with 5% native PAGE, stained with GelRed. **B** Schematic diagram of optimized tool for sample loading. **C** An open-end-thin-walled polypropylene 5 mL centrifuge tube from Beckman Coulter.**D** Beckman Coulter MLS-50 Swinging-Bucket Rotor. **E** Beckman Coulter Optima MAX-XP Benchtop ultracentrifuge

Equimolar ratios of histones H2A, H2B, H3, and H4 were mixed and dialyzed together to get rid of denaturants and form the histone octamer. The octamer was purified by size-exclusion chromatography. Then, the purified octamer, the 167 bp DNA fragment, and histone H1.4 were combined to assemble the target complex H1.4-NCP^167^ by reducing the salt concentration via dialysis (Fig. 3A).

**[?TROUBLESHOOTING NO.1]**

### The design of the solid-state NMR rotor loading tool

As highlighted in the introduction, the success of sampling filling in solid-state NMR spectroscopy analysis hinges on using a specialized MAS rotor loading tool. In this paper, we present a novel rotor loading tool. Figure 3B showcases the conceptual design of the loading tool, which can be conveniently placed directly into the centrifuge basket. The tool is shaped to fit inside the centrifuge basket, with a groove at the bottom adjusted to the size of the rotor.

Once the rotor is inside the loading tool, a solution-state sample can be added to the upper funnel. After ultracentrifugation, the isotopically labeled biomolecular sample can be loaded into the rotor in solid form. Finally, carefully remove the supernatant from the funnel, remove the rotor, and attach the cap.

The design drawings of the ssNMR rotor loading tool were created using AutoCAD software, while the three-dimensional model was developed using SolidWorks. The tool is machined from polyoxymethylene (POM), commonly known as "Delrin", a crystalline plastic renowned for its rigidity. POM exhibits excellent geometric stability, impact resistance, fatigue resistance, creep resistance, wear resistance, and heat resistance, even at low temperatures. It is also not prone to moisture absorption and has a specific gravity of 1.42 g/cm^3^ and a heat distortion temperature of 172 °C.

The loading tool has a centrifuge tube appearance, with the external profile identical to that of the 5-mL open-top thin-walled polypropylene centrifuge tube from Beckman Coulter (Fig. 3C), making it compatible with the MLS-50 Swinging-Bucket Rotor from Beckman Coulter (Fig. 3D). The tool can be used with the Optima MAX-XP Benchtop Ultracentrifuge from Beckman Coulter (Fig. 3E) for high-speed centrifugation to load samples. The temperature setting range is 0–40 °C, with commonly used temperatures for biological samples between 4 °C and 25 °C. The centrifugal force setting range is 3000–268,000 *g*, with the tool typically set to 240,000 *g*.

**[TIP]** The ssNMR rotor loading tool is designed for use with a swinging-bucket centrifuge, not a fixed-angle one. Using a fixed-angle ultracentrifuge with the tool can lead to damage due to the misalignment of the direction of centrifugal force and the long axis of the loading tool.

**Figure 4:**
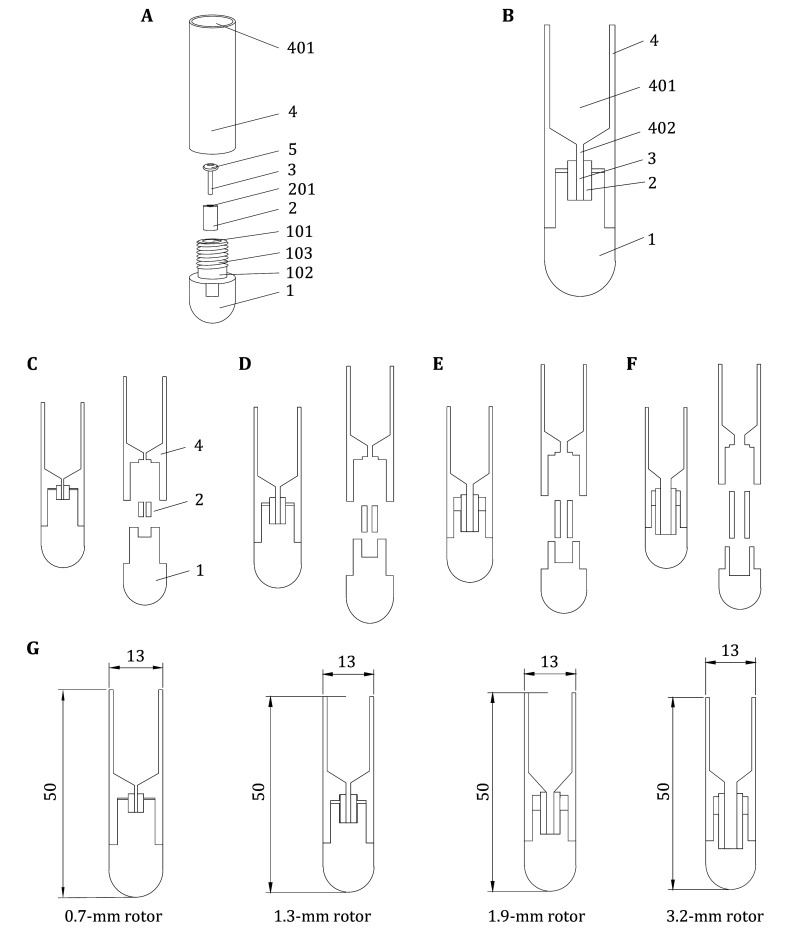
**A** Exploded view of 1.3-mm rotor sampling tool. **B** Cutaway drawing of 1.3-mm rotor sampling tool. **C** Plan of 0.7-mm sampling tool for ssNMR. **D** Plan of 1.3-mm sampling tool for ssNMR. **E** Plan of 1.9-mm sampling tool for ssNMR. **F** Plan of 3.2-mm sampling tool for ssNMR. **G** The size of the in-house tools

Figures 4A and 4B present the rotor loading tool's de tailed design, comprising a base (1), a fixing piece (2), and the main body (4), which is specifically designed for this purpose. The base's top (1) is concave downward to create a mounting groove (101), which holds the fixing piece (2) that has a pinhole (201) for rotor (3) installation. Removing the fixing piece (2) allows visual inspection of the installation groove's contact surface with the rotor (3) and fixing piece (2) to ensure a flat surface, minimizing the risk of the rotor (3) damage. The main body (4) contains the sample solution with a funnel-shaped bottom. The bottom of the solution space (401) has a perforated hole (402) along its axial direction. The bottom of the main body (4) connects to the top of the fixing piece (2) and the base's top (1), both of which are sealed. The perforated hole (402) is coaxially aligned with the pinhole (201). After centrifuging the solution, the pellets enter the rotor (3) through the pinhole (402). There is one O-ring (5) between the rotor (3) and perforated hole (402) to seal the solution.

In practice applications, the rotor (3) can be placed in the loading tool, and the target biomolecules in solution can be directly collected into the solid-state NMR MAS rotor via ultracentrifugation, enabling a one-step process from solution or suspension to sample loading. This process reduces sample loading steps, simplifies operation, and minimizes the loss of high-cost isotope-labeled samples during transfer and loading, thus saving the experimental costs. Moreover, it ensures the activity of biological samples and prevents sample dehydration.

The design covered in this paper provides four sets of loading tools suitable for the MLS-50 Swinging-Bucket rotor, all of which have been granted patent No. CN202320115093.7 protection (Zhang *et al.* 2023), and are used to load samples for the 0.7-mm rotor, the 1.3-mm rotor, the 1.9-mm rotor, and the 3.2-mm rotor, respectively, as shown in Figs. 4C–4F, in that order. The tool dimensions specifically applicable to the 0.7, 1.3, 1.9, and 3.2-mm rotors are shown in Fig. 4G.

**[TIP]** The shape of this series of solid-state NMR rotor loading tools can be adjusted according to the ultracentrifuge and centrifugal rotor model. The loading tools mentioned in this paper are adapted to the Beckman Coulter MLS-50 Swinging-Bucket Rotor, which can be used to load samples in ultracentrifugation on the Optima MAX-XP Benchtop Ultracentrifuge for sedimentation NMR experiments.

### Collecting nucleosome complexes via sample loading tool in a high-speed centrifuge

#### Assemble the sample loading device

**Figure 5:**
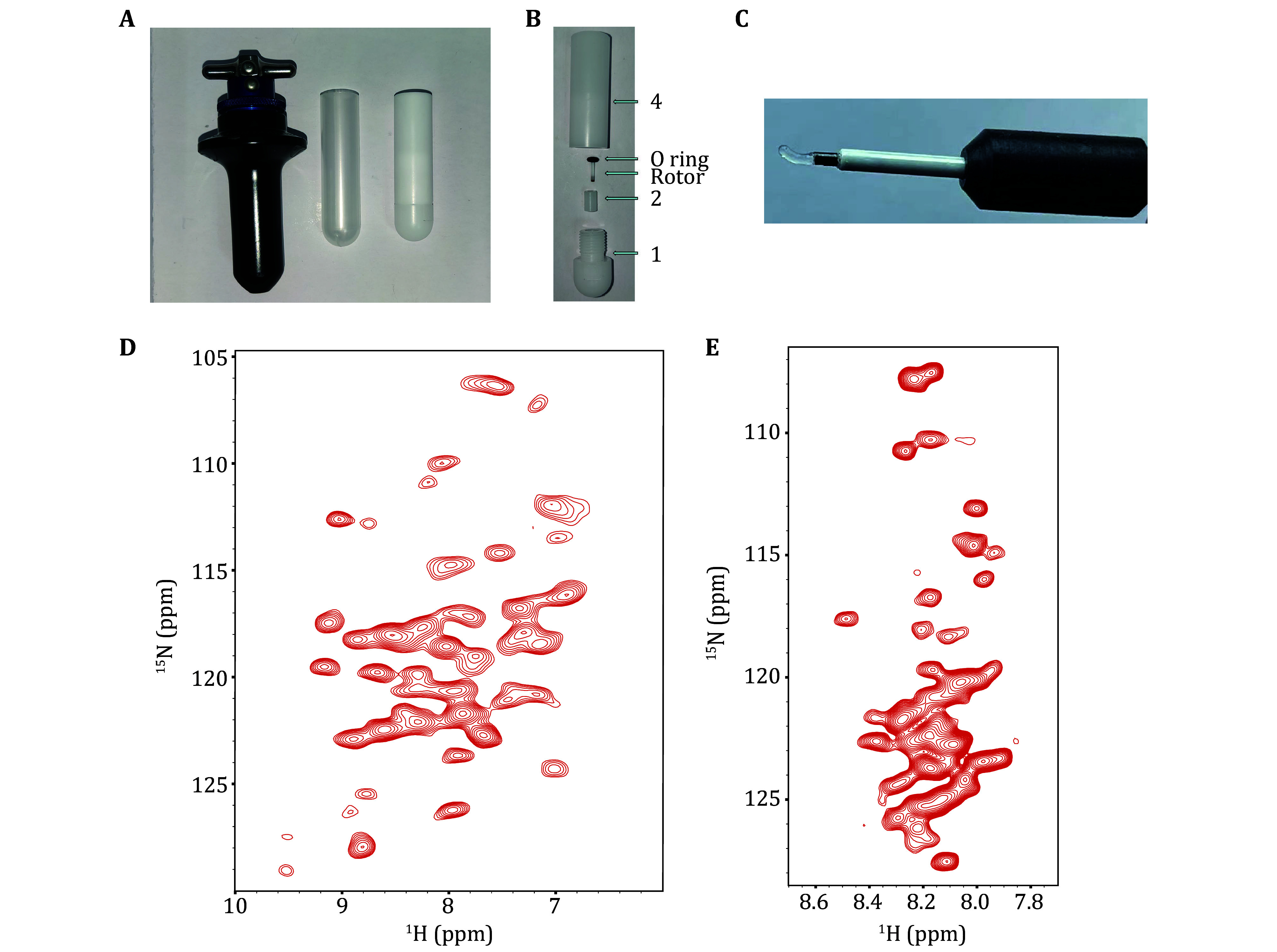
**A** Pictures displayed in Beckman Coulter MLS-50 Swinging-Bucket Rotor (left), a polypropylene centrifuge tube (13 × 51 mm) (middle), a sample loading tool of 1. 3-mm ssNMR rotor (right). **B** Physical display of the disassembly of the sampling tool with a 1.3-mm ssNMR rotor and O ring. **C** The transparent gel is the H1.4-NCP^167^ in a 1.3-mm ssNMR rotor obtained by ultracentrifugation sedimentation with a 1.3-mm ssNMR rotor loading tool. **D** CP-based 2D ^1^H-^15^N correlation spectrum of H1.4 (MAS = 55 kHz, Tset = 230K). **E** INEPT-based 2D ^1^H-^15^N HSQC spectrum of H1.4 (MAS = 60 kHz, Tset = 245K)

To assemble the sample loading device, place fixing piece (2) into the mounting groove (101) of base (1) and insert the rotor (3) into the pinhole (201) in fixing piece (2). Secure the rotor (3) in place by using an O-ring (5) with a suitable size above the fixing piece (2). Connect the O-ring (5)-rotor (3)-fixing piece (2)-base (1) assembly to the main body (4) by rotating it until securely tightened. With this, the sample loading device assembly is complete. The images of the 1.3-mm rotor loading tool can be found in Figs. 5A and 5B.

**[TIP]** The O-ring is for one-time use only and cannot be reused. A used O-ring may undergo irreversible deformation due to compression and prolonged high-speed centrifugation. Therefore, a new suitable O-ring must be used each time to ensure the proper cushioning and sealing effects.

**[?TROUBLESHOOTING NO.2]**

#### Load the sample into the tools

Add approximately 700 μL of complex H1.4-NCP^167^ in solution form (measured DNA concentration of 2.3 mg/mL) to the funnel at 401 of the assembled 1.3-mm rotor loading tool main body (4). Weigh the bucket with an analytical balance, ensuring that the difference in weights of the leveling is no more than ±0.1 mg. Place the leveled bucket into the MLS-50 rotor and ensure that the bucket lid and body numbers match, that the lids are attached tightly, and that the buckets are attached correctly to the rotor and leveled in pairs (1 and 3, and 2 and 4 are leveled). Check that the bucket numbers match the MLS-50 marking position before placing the rotor in the ultracentrifuge. Figures 5A and 5B illustrate the MLS-50 swinging-bucket rotor and the assembled 1.3- mm rotor loading tool.

**[TIP]** For proper sample loading, the sample volume transferred into the tool should be controlled and adjusted according to the volume of the main body (4). Generally, higher sample concentrations result in more effective sample sedimentation. If the sample is collected at 4 °C, pre-cool the ultracentrifuge and rotor. For accurate results, perform rigorous leveling and repeat the process twice for verification.

#### The centrifugation process

After all checks are completed, carefully place the rotor into the chamber of the ultracentrifuge, gently rotate the rotor, and close the centrifuge lid when a click is heard. On the operation page of the ultracentrifuge, select the rotor type MLS-50 and set the parameters for high-speed centrifugation: centrifugal force 240,000 *g*, time 60 h, temperature 4 °C. Click the vacuum button to start vacuuming. Once the vacuum level drops below 100 μmHg and the temperature reaches the set temperature, click start to begin high-speed centrifugation. At this time, the background of the operation page will change from blue to green. Do not leave until the speed stabilizes and reaches the set speed. Always pay attention to the centrifuge when it is running.

**[TIP]** During acceleration, the vacuum level may rise and then fall. When the speed stabilizes, and the centrifuge operates properly, the vacuum level should be close to 0 μmHg.

Once the centrifugation process is complete and the rotor speed has decreased to 0, the operation page background will change from green to blue. To release the vacuum, click the vacuum button and wait for the centrifuge to make a hissing sound as the air is released. Once the hissing sound stops, open the centrifuge lid and carefully remove the centrifuge rotor, placing it on the rotor adapter stand. Close the centrifuge lid, click the vacuum button to vacuum for 10–20 s, and then turn off the centrifuge.

Next, remove the sample bucket, open the lid, and carefully remove the supernatant from the funnel. Loosen the assembly threads (O Ring-rotor-fixing-piece-base) from the main body (4), disassemble the tool, and take out the rotor. Use a magnifying glass to check if the rotor is damaged, and use the Bruker-provided sample pestle with etched lines to measure the sample loading volume, and carefully cover the rotor cap. The typical sample state should be a transparent gel (Fig. 5C).

**[TIP]** When removing the supernatant from the funnel, it is crucial to aspirate slowly and carefully remove most of the supernatant. If aspiration is too vigorous, it can disturb the sample in the rotor. When capping the solid-state NMR rotor, use the Bruker-supplied rotor tool and proceed cautiously to avoid damaging the propeller wings on the cap.

**[?TROUBLESHOOTING NO.3 & NO.4]**

#### Spinning stability test

Put the solid-state NMR rotor with the cap closed into the MAS test bed for the spinning stability test.

**[TIP]** Before conducting solid-state NMR experiments, it is essential to thoroughly examine the rotor for any deformations or scratches, particularly on the caps and bottoms. This examination is critical to prevent potential rotor crush during experiments.

### The ssNMR spectra of the complex H1.4-NCP^167^

Place the 1.3-mm NMR rotor containing nucleosome complex H1.4-NCP^167^ into a 600 MHz solid-state NMR spectrometer. Here protein H1.4 is ^13^C, ^15^N, and ^2^H labelled.

With the new ultracentrifugation solid-state NMR rotor packing tool, we obtained proton-detected solid-state NMR spectra of ^13^C, ^15^N, ^2^H-labelled H1.4 assembled in unlabelled 167 NCP in complex H1.4-NCP^167^ with a 1.3-mm MAS rotor. The experiments were conducted at MAS 55 kHz for the CP-based 2D ^1^H-^15^N correlation spectrum and at 60 kHz for the INEPT-based 2D ^1^H-^15^N HSQC spectrum, respectively. Given the good resolution of the 2D spectrum, higher dimensional solid-state NMR experiments can be performed to obtain backbone assignments (Figs. 5D and 5E).

**[?TROUBLESHOOTING]**

Troubleshooting is shown in Table 3.

**Table 3:** Troubleshooting table

NO.	Problems observed	Possible reasons	Solutions
1	The complex H1.4-NCP^167^ may be contaminated with free NCP^167^	Insufficient amount of H1.4	Adjust the ratio of H1.4 to NCP^167^ and increase the amount of H1.4
2	Liquid leakage sometimes occurs. Liquid is leaking from the tool into the rotor chamber of the bucket	(a) Mismatch of O Ring used, or O Ring reused, not sealing (b) The connection between the base (1) combination part and the main body (4) is not tightened	(a) A suitable O Ring is used for each size of the tool and is for one-time use. (b) Tighten the threads between the base (1) to the main body (4)
3	Wear on the bottom of the NMR rotor	(a) Failure to place the rotor vertically into the fixing piece (2) (b) The contact surface of the mounting groove (101) with the rotor (3) and fixing piece (2) is not flat	(a) Place the rotor vertically into the piece (2) (b) Replace the base 1 when the uneven contact surface is detected
4	No or insufficient sample in the rotor	(a) Insufficient amount of sample placed in the tool (b) The centrifugal force is not high enough, or the centrifugation time is not long enough (c) After centrifugation, vigorous aspiration of the funnel supernatant, resulting in suction of the sample out of the rotor	(a) Prepare a sufficient amount of sample to be loaded into the tool (b) Increase the centrifugal force and centrifugation time to ensure effective loading. Calculations based on the respective centrifuge's configuration and target molecular weight are recommended. (c) Slowly aspirate the supernatant from the main body (4) and remove most of it after centrifugation

### MATERIALS

The main drugs and reagents used in the experiment are detailed in Table 4.

**Table 4:** Drugs and reagents

Name	Manufacturer
KH_2_PO_4_	Sinopharm Chemical Reagent Co., Ltd
NaOH	Sinopharm Chemical Reagent Co., Ltd
Deuterium oxide (D,99.9%)	Cambridge Isotope Laboratories
D-Glucose (U-13C6,99%)	Cambridge Isotope Laboratories
Ammonium Chloride (^15^N,99%)	Cambridge Isotope Laboratories
MgSO_4_	Sinopharm Chemical Reagent Co., Ltd
CaCl_2_	Sinopharm Chemical Reagent Co., Ltd
Tris-HCl	Diamond
KCl	Sinopharm Chemical Reagent Co., Ltd
NaCl	Sinopharm Chemical Reagent Co., Ltd
HEPES	Solarbio
EDTA	Sangon Biotech
DTT	Sangon Biotech
Glycerol	KESHI
IPTG	Sangon Biotech
Trans 2K DNA Marker	Trans
Unstained Protein MW Marker	Thermo

The main instruments and equipment used in the experiment are detailed in Table 5.

**Table 5:** Instruments and equipment

Name	Model	Manufacturer
FPLC (Fast Protein Liquid Chromatography)	AKTA Pure 29-0182-24	Cytiva
HPLC (High Performance Liquid Chromatography)	LC-20AT	Shimadzu
Mini Centrifuge	EQ-6K	Eastwin Scientific Equipmentsinc
Centrifuge	Microfuge16	Beckman Coulter
Desktop refrigerated microcentrifuge	Microfuge20R	Beckman Coulter
Benchtop refrigerated centrifuge	Allegra X-15R	Beckman Coulter
High-Speed Refrigerated Centrifuge	AvantiJ-26SXP	Beckman Coulter
Benchtop Ultracentrifuge	Optima MAX-XP	Beckman Coulter
Swinging-Bucket Rotor	MLS-50	Beckman Coulter
Analytical and Precision Balances	Practcum	Sartorius
Spectrophotometer	DS-11+	DeNovix
Gel Documentation and Analysis	GenoSens 2100	Clinx Science Instruments Co., Ltd.
Ultrasonic Cell Disruptor	Scientz-IID	Ningbo Scientz Biotechnology Co., Ltd
JN-Mini Pro Low-temperature Ultra-high pressure cell disrupter	JN-Mini Pro	JNBIO
SC Submarine Horizontal Electrophoresis System	Mini SC	CAVOY
Vertical Electrophoresis System	Mini P-4	CAVOY
Lyophilizer	L3-65	Capable
Peristaltic Pump	BT100-2J	Longerpump
O Ring	Outer diameter 4*1.5 mm	Ojp Hardware Flagship Store
ssNMR Rotor	1.3 mm	Bruker
600 MHz magnetic resonance spectrometer	Bruker Avance Neo WB 600 MHz	Bruker

## Conflict of interest

Fan Shi, Tong Zhang, Juan Li, Chaowei Shi and Shengqi Xiang declare that they have no conflict of interest.
